# Treatment of Unruptured Vertebral Artery Aneurysm Involving Posterior Inferior Cerebellar Artery With Pipeline Embolization Device

**DOI:** 10.3389/fneur.2021.622457

**Published:** 2021-06-10

**Authors:** Weiqi Fu, Huijian Ge, Gang Luo, Xiangyu Meng, Jiejun Wang, Hengwei Jin, Youxiang Li

**Affiliations:** ^1^Department of Interventional Neuroradiology, Bejing Neurosurgical Institute and Beijing Tiantan Hospital, Capital Medical University, Beijing, China; ^2^Department of Neurosurgery, Fushun Central Hospital, Fushun, China

**Keywords:** pipeline embolization device, vertebral artery, posterior circulation aneurysms, dissecting aneurysms, pica

## Abstract

**Background:** Treatment of unruptured vertebral artery aneurysm involving posterior inferior cerebellar artery (PICA) is challenging. The experience of pipeline embolization device (PED) therapy for these lesions is still limited.

**Objective:** To evaluate the safety and efficacy of the PED for unruptured vertebral artery aneurysm involving PICA.

**Methods:** Thirty-two patients with unruptured vertebral artery aneurysm involving PICA underwent treatment with PED were retrospectively identified. Procedure-related complications, PICA patency, clinical, and angiographic outcomes were analyzed.

**Results:** Thirty-two aneurysms were successfully treated without any procedure-related complications. Images were available in 30 patients (93.8%) during a period of 3–26 months follow-up (average 8.4 months), which confirmed complete occlusion in 17 patients (56.5%), near-complete occlusion in 9 patients (30%), and incomplete occlusion in one patient (3.3%). Parent artery occlusion (PAO) was occurred in 3 patients (10%). Twenty-eight of 30 PICA remained patent. The two occlusions of PICA were secondary to PAO. At a mean of 20.7 months (range 7–50 months) clinical follow-up, all the patients achieved a favorable outcome without any new neurological deficit.

**Conclusion:** PED seems to be a safe and effective alternative endovascular option for patients with unruptured vertebral artery aneurysm involving PICA.

## Introduction

Vertebral artery aneurysm account for 11% of posterior circulation aneurysms and 3–5% of subarachnoid hemorrhage (SAH) (1–3). The outcome of ruptured vertebral artery aneurysm is dismay with a high mortality rate of up to 50% (4). Therefore, it is necessary to take more aggressive treatment for the unruptured vertebral artery aneurysm considering the risk of progression and rupture, especially vertebral artery aneurysm involving posterior inferior cerebellar artery (PICA). Previous study reported that PICA involvement is risk factor of progression (5), and it has the highest morbidity among the ruptured vertebral artery aneurysm (6). However, Treatment of vertebral artery aneurysm involving PICA is challenging. The major consideration including preservation of the PICA and complete occlusion of the aneurysm (7). Selection of appropriate treatment modalities for these lesions remains controversial, the most acceptable conventional endovascular methods include internal coil trapping with revascularization of PICA and stent-assisted coiling. However, each method has its own drawback and limitation in terms of retaining the patency of PICA (8). PED has emerged as a popular treatment option for intracranial aneurysms and achieved promising results (9). This device can preserve parent vessels as well as major side branches while excluding aneurysm from circulation by disrupting flow within the aneurysm and remodeling vessel (10). These characteristics may be suitable for the treatment for vertebral artery aneurysm involving PICA. However, few articles have been published describing patient outcome following PED in treatment of these lesions. In this study, we report our experience regarding the efficacy and safety of the treatment of unruptured vertebral artery aneurysm involving PICA with PED.

## Methods

### Patients

In accordance with our institutional review board, a retrospective study by extracting patient data from a prospectively maintained database was performed. Three hundred and twenty-four patients with vertebral artery aneurysms treated with endovascular methods in our institution between January 2016 and October 2019 were reviewed. Cases were excluded if the aneurysm did not involve PICA, the patients presented with subarachnoid hemorrhage, or the aneurysms were not treated with PED. Thirty-two patients with unruptured vertebral artery aneurysm involving PICA and using PED as treatment modality were included. Every patient was discussed by at least 3 experienced senior interventional radiologists for indications, which include high risk of rupture and uncontrolled progressive clinical symptoms (headache, dizziness, ataxia, vomiting, dysphagia; Table 1). All the patients provided written informed content and the study was approved by ethics committee of our institution.

**Table 1:** Basic information and aneurysm characteristics.

**Characteristics**	**No./Ave (range)**	**%/SD**
Patients	32	
Aneurysms	32	32
Genders (males)	23	71.9%
Age (years)	52 (17–67)	±9.37
**Risk factors**		
Hypertension	14	43.8%
Diabetes	4	12.5%
Smoking	7	21.9%
**Presentation**		
Headache	14	43.8%
Dizziness	6	18.8%
Ataxia	2	6.3%
Dysphagia	2	6.3%
Vomiting	2	6.3%
Incidence	6	18.8%
**Aneurysm morphology**		
Fusiform	23	71.9%
Saccular	9	28.1%
Length (mm)	13.9 (7–27)	±5.18
Width (mm)	8.9 (4-18)	±3.67
**VA dominance**		
Co-dominant	20	62.5%
Right	9	28.1%
Left	3	9.4%
**Baseline MRS number (%)**		
MRS (0–2)	32	100%
MRS (3–5)	0	0

*SD, Standard deviation; Ave, Average; VA, Vertebral artery; MRS, Modified Rankin Scale*.

### Procedure

All endovascular procedures were performed under general anesthesia and systemic heparinization was administered after placement of the sheath, a 6-F guiding catheter (Codman, Raynham, Massachusetts, USA) was placed in the distal V2 segment. Using a coaxial system, Marksman (EV3, Irvine, California, USA) was navigated over a 0.014-inch microwire in the target artery beyond the aneurysm. Once the PED reached scheduled position, it was released carefully by a combination of withdrawing the Marksman catheter and advancing the delivery wire. For the aneurysms with necessity of adjunctive coil embolization, additional coiling was conducted through a pre-jailed Echelon-10 catheter (EV3, Plymouth, Minnesota, USA) to loosely pack the aneurysmal sac. Coil embolization was performed to facilitate the thrombosis of the aneurysm, instead of completely occlude the aneurysm. A final angiogram was obtained after deployment to assess stent placement and vessel patency. Cone-beam CT was performed to ascertain the wall apposition of the device.

### Antiplatelet and Anticoagulation Treatment

Patients were pre-medicated with a dual antiplatelet regimen (75 mg of clopidogrel and 100 mg of aspirin daily) for at least 5 days before treatment. During the procedure, we administered an intravenous bolus dose of heparin (70 IU/kg) and continued heparinization to maintain an activated clotting time throughout the procedure of two to three times greater than the baseline value. Dual antiplatelet therapy was continued for 6 months after the procedure, and aspirin was continued indefinitely to prevent thrombi forming in the stents.

### Outcome Management

Clinical outcome was measured by the Modified Rankin Scale (MRS) at the latest available follow-up. Angiographic outcomes were determined by digital subtraction angiography (DSA)or computed tomographic angiography (CTA) which was scheduled at 3–6 months and 1–2 years postoperatively. A follow-up MRS scores of 0–2 were defined as a favorable outcome; MRS scores of 3–6 were considered a poor outcome. Aneurysms occlusion at follow- up was categorized as complete (100%), near-complete (≥90%) or incomplete (<90%). Follow-up angiographic images were also reviewed for patency of PICA and parent vessels.

### Statistical Analysis

Since the number of patients is relatively small and there is no sub-group analysis in the study, the data were presented in descriptive methods. For continuous variables, data that obeyed normal distribution are presented as mean and standard deviation. Data that did not obey normal distribution are presented as median and inter-quartile range. For categorical variables, data are presented as the absolute value followed by percentage.

## Results

### Patient and Aneurysm Characteristics

A total 32 patients with vertebral artery aneurysm involving PICA were included in the present study. The mean age was 52 years old (range 17–67 years old). This study had greater proportion of males (71.9%) compared with that of females (28.1%). Patients risk factors include hypertension (43.8%), diabetes (12.5%) and smoking (21.9%). Of the 32 patients, twenty-six presents various clinical symptoms including headache (43.8%), dizziness (18.8%), ataxia (6.3%), dysphagia (6.3%), and vomiting (6.3%). The other six were incidentally identified without any symptoms.

The mean length and diameters of aneurysms were 13.94mm (Ranges from 7 to 27 mm) and 8.88 mm (Ranges from 4 to 18 mm), respectively. In terms of morphology, the majority of cases were fusiform (71.9%) compared to saccular (28.1 %). In terms of VA dominance, the majority of cases were codominant (62.5%) compared to single dominant (37.5%). All patients and aneurysm characteristics were summarized in Table 1.

### Aneurysm Management

Patients with aneurysms of an irregular morphology and clinical symptoms (headache, dizziness, ataxia, vomiting, and dysphagia) were defined as having indications for treatment. All aneurysms were treated successfully with endovascular reconstruction including PED alone (*n* = 28) and pipeline-assisted coiling (*n* = 4). Thirty-three PEDs were used in 32 procedures. Thirty-one cases were treated with one PED, and double PEDs were used in one patient. Balloon angioplasty was used in one patient with parent artery stenosis. The time of contrast within aneurysms increased moderately in all the patients after PED placement. The aneurysm, parent artery and stent details are listed in Table 2.

**Table 2:** Patients, aneurysm and stent details.

**Patients**	**Gender/Age (years)**	**Morphology of AN**	**Size of AN (mm)**	**Diameter of parent artery (Proximal/Distal of AN, mm)**	**Size of stent (mm)**
1	Male/58	Fusiform	11*20 mm	3.7/3.2 mm	4.0*35
2	Male/51	Fusiform	9*12 mm	3.3/3.1 mm	3.5*30
3	Female/54	Fusiform	6*15 mm	3.4/3.3 mm	3.5*20
4	Male/55	Fusiform	8*15 mm	3.3/2.8 mm	3.75*35
5	Male/54	Fusiform	6*11 mm	3.2/3.1 mm	3.5*30
6	Male/52	Saccular	18*17 mm	4.3/3.9 mm	4.5*35
7	Female/47	Fusiform	8*12 mm	3.4/3.1 mm	3.5*30
8	Male/55	Fusiform	8*12 mm	3.3/2.9 mm	3.5*30
9	Female/54	Saccular	15*17 mm	3.6/3.3 mm	4.0*35
10	Female/30	Saccular	5*7 mm	3.3/3.0 mm	3.5*20
11	Female/55	Saccular	11*12 mm	3.6/3.1 mm	3.75*25
12	Female/67	Saccular	7*9 mm	3.3/3.0 mm	3.5*30
13	Female/44	Saccular	8*10 mm	3.2/3.1 mm	3.5*30
14	Female/54	Fusiform	4*8 mm	3.8/3.1 mm	4.0*25
15	Male/52	Saccular	8*10 mm	4.1/3.5 mm	4.5*25
16	Male/53	Fusiform	9*13 mm	4.3/3.8 mm	4.5*25
17	Male/51	Fusiform	20*27 mm	4.8/3.7 mm	5.0*35
18	Male/61	Fusiform	7*14 mm	3.5/3.2 mm	3.75*35
19	Female/53	Fusiform	5*8 mm	3.3/3.2 mm	3.5*25
20	Male/67	Fusiform	7*12 mm	3.7/3.5 mm	4.0*35
21	Male/64	Fusiform	9*17 mm	3.7/3.2 mm	4.0*35
23	Male/56	Fusiform	5*12 mm	4.1/3.7 mm	4.25*25
24	Female/56	Fusiform	8*15 mm	3.4/3.1 mm	3.5*30
25	Male/17	Fusiform	9*12 mm	3.5/3.1 mm	3.75*35
26	Male/54	Fusiform	9*21 mm	3.3/3.1 mm	3.5*25
27	Male/46	Fusiform	7*18 mm	4.0/3.5 mm	4.25*35
28	Male/46	Saccular	6*8 mm	3.1/2.9 mm	3.25*35
29	Male/55	Saccular	6*8 mm	4.1/4.2 mm	4.25*35
30	Male/55	Fusiform	10*21 mm	4.3/4.0 mm	4.5*35/4.0*30
31	Male/53	Fusiform	11*17 mm	3.3/2.9 mm	3.5*25
31	Male/46	Fusiform	12*18 mm	3.9/4.5 mm	4.75*30
32	Male/52	Fusiform	7*14 mm	3.4/3.1 mm	3.5*30

*AN, Aneurysm*.

### Complications

There was no intra-procedural complication. Three patients developed delayed complication (parent artery occlusion PAO) during follow up which did not lead to any neurological symptoms or neurological deficits.

### Angiographic and Clinical Follow-Up Outcome

**Figure 1:**
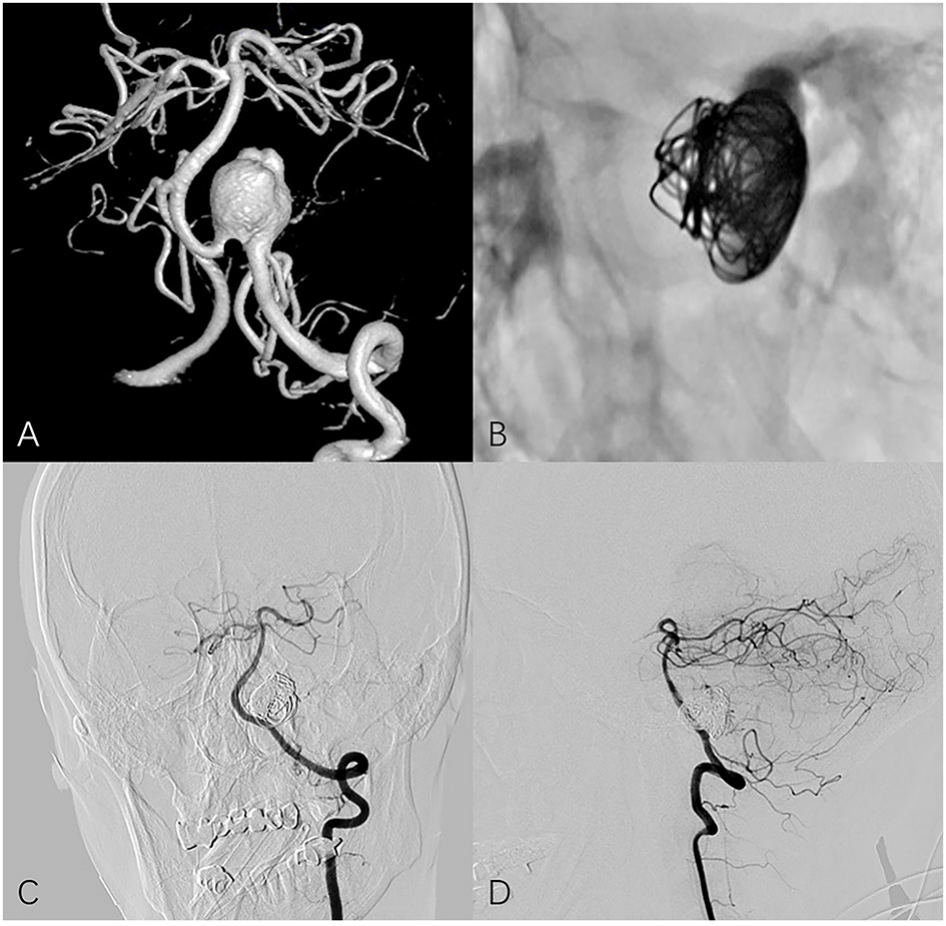
Fifty four year-old female with left vertebral artery aneurysm. **(A)** A 3-dimensional reconstruction image of a vertebral angiogram shows an irregular aneurysm involving PICA. **(B)** Non-subtracted oblique view shows PED in place and loosely filling coils. Follow-up frontal **(C)** and lateral **(D)** angiography shows the aneurysm is completely occluded and PICA remains patency.

**Figure 2:**
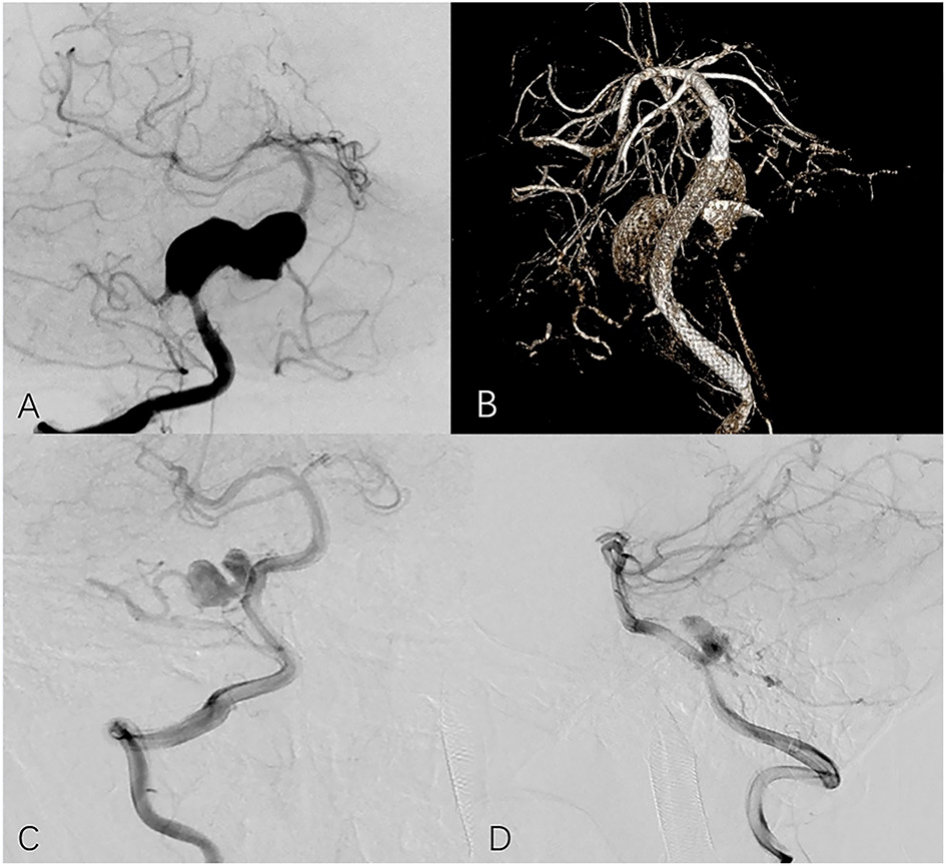
Seventeen year-old male with right vertebral aneurysm extending to basilar artery. **(A)** Preoperative frontal angiogram shows a giant vertebrobasilar aneurysm involving PICA. **(B)** Postoperative DynaCT indicates good opening of the PED and apposition. The 13-month follow-up frontal **(C)** and lateral **(D)** angiogram shows the aneurysm is incompletely occluded and the PICA is preserved.

**Figure 3:**
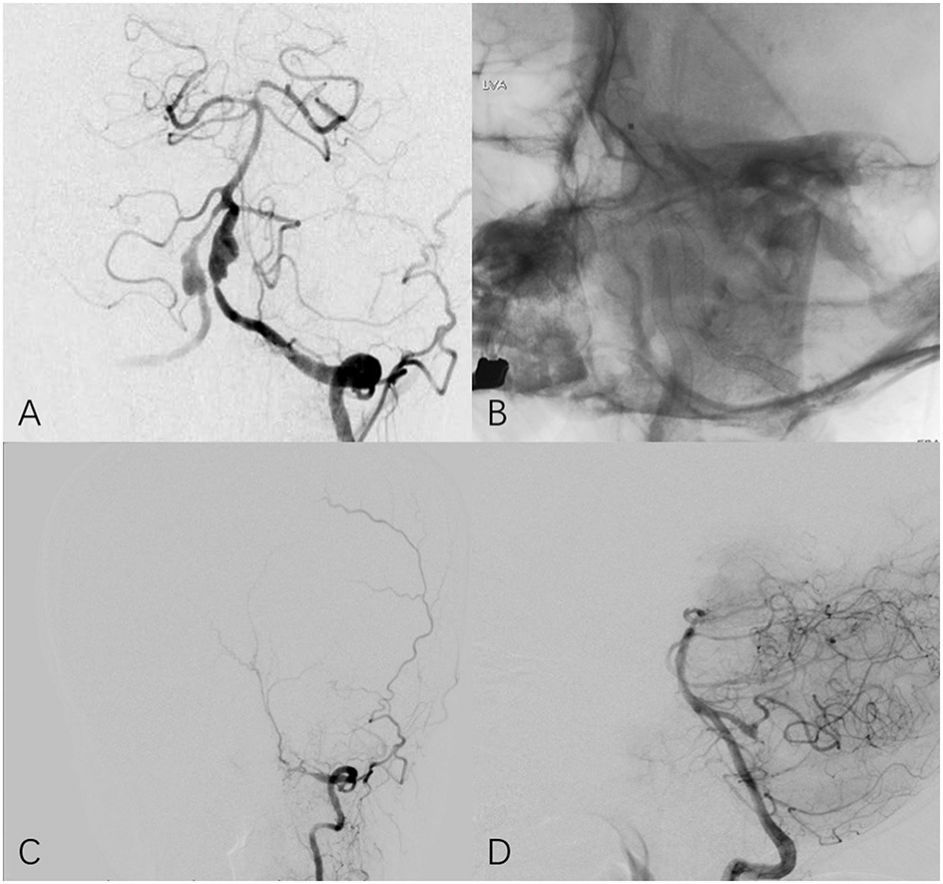
Fifty four year-old female with bilateral vertebral aneurysm. **(A)** Frontal angiogram of the left vertebral artery shows bilateral dissecting aneurysm and the left involved PCIA. **(B)** Non-subtracted frontal view shows bilateral PED in place. **(C)** The 25-month follow-up left **(C)** and right **(D)** angiogram indicates left vertebral artery is occluded at V3 segment and PICA remains patency through contralateral circulation.

Of the 32 patients, follow-up DSA or CTA was available in 30 patients. The average angiographic time was 8.3 months (3–26 months) after procedure. Seventeen (56.7%) of follow-up cases were completely occluded angiographically including four patients with adjunctive coiling (Figure 1). Nine (30%) cases were near completely occluded, and one case (3.3%) was incompletely occluded (Figure 2). Parent artery occlusion occurred in 3 (10%) cases. Involved PICA remained patency in 28 of 30 patients including one of PAO with retrograde filling from contralateral VA (Figure 3). The other two patients with PAO showed PICA occlusion. Clinical follow-ups were available in all 32 patients. the mean time of follow-up was 20.7 months (7–50 months). All patients achieved favorable outcome at the last follow-up. There were no morbidity and mortality. All follow-up outcomes were summarized in Table 3.

**Table 3:** Procedural details and follow-up outcomes.

**Items**	**Number (100%)**
Procedural success	32 (100%)
PED numbers	33
1	31 (96.9%)
2	1 (3.1%)
Adjunct coil deployment	4 (12.5%)
Balloon angioplasty	1 (3.1%)
Imaging follow-up available	30 (93.8%)
**Follow-up modality**	
Digital subtraction angiography (DSA)	20 (66.7%)
Computed tomographic angiography (CTA)	10 (33.3%)
**Follow-up occlusion rate**	
Complete occlusion	17 (56.7%)
Near-complete occlusion	9 (30%)
Incomplete occlusion	1 (3.3%)
Patency of PICA	28 (93.3%)
**Delay complications**	
PAO	3 (10%)
Clinical follow-up available	32 (100%)
**Follow-up MRS**	
0–2	32 (100%)
3–6	0 (0%)

*PED, Pipeline embolization device; PICA, Posterior inferior cerebellar artery; PAO, Parent artery occlusion; MRS, Modified Rankin Scale*.

## Discussion

PICA is an important branch originate from intracranial VA, which supplies part of cerebellum and medulla. The sacrifice of PICA will result in severe ischemic stroke and poor clinical outcome. Studies have shown that ischemic complications happened in 21.7% cases in which the PICA is sacrificed (8). When vertebral artery aneurysm involves the ostium of PICA, the treatment become complicated and challenging and the options are also limited (11). One consideration is to maintain the PICA patency. Various techniques were utilized for this purpose including loosely filling aneurysm, stent placement from VA to PICA, and surgical bypass using an occipital artery (OA)-to-PICA, PICA side-to-side anastomosis or PICA transposition. However, each approach has its own limitation and is unable to be an ideal and routine method. Loosely filling when using stent-assisted coiling result in high rate of incomplete occlusion which is thought to be an important factor to predict the postprocedural recurrence. A previous study for vertebral artery aneurysm treated with stent assisted coiling found 18% vertebral artery aneurysm involving PCIA experienced recurrence, whereas only 5% occurred in those without PICA involvement (12). The uncertainty of releasing a stent in a vessel <2 mm diameter and the difficulty of introducing a catheter into PICA due to the disadvantaged angel made the using of stent placement from VA to PICA only for a few selective patients (13, 14). Surgical bypass in posterior circulation is technically challenging and may require a lengthy operative time. Furthermore, bypass associated with various of surgical complications such as lower cranial nerve palsy and anastomosis failure (15). A review conducted by Chen et al. (16) noted two graft occlusion and three infarction occurred in 19 patients with vertebral artery aneurysm involving PICA treated by bypass, suggesting this method has been typically reserved for the cases not amendable to endovascular treatment. In the present study, all the PEDs were successfully deployed without technical events. one PED was used in thirty-one patients (96.9%) and the exceptional case (3.1%) using two devices harbored a giant vertebral artery aneurysm extending to basilar artery. For the four large saccular aneurysms, one or two coils were used to facilitate the thrombosis of the aneurysm instead of intensely coiling. Of the 30 patients with follow-up imaging, 28 remained the patency of PICA and two occurred PICA occlusions secondary to PAO. Mazur et al. (17) reported that all the PICA with origin covered by PED remained patency in eight patients and suggested when appropriately sized to the vessel wall and positioned in the VA, the device may cover the origin of PICA without impairing flow through the branching artery. Levitt et al. (18) reported that all the PICA maintained patency in six cases with vertebral artery aneurysms involving PICA treated by flow diverting stent. Similar results were reported by Adeeb et al. (19) and Wu et al. (20). These data suggested that the PICA can be effectively and safely preserved when using PED in treatment of these lesions. Additionally, in terms of conserving PICA, PED appears to be more convenient than conventional endovascular methods.

Nearly 86.7% of patients with angiographic follow-up achieved complete or nearly complete occlusion at mean 7.8 months follow-up without recurrence and retreatment. The only one incomplete occlusion (3.3%) was occurred in a patient with a giant vertebral artery aneurysm extending to basilar artery which was substantially attenuated. In addition, complete occlusion also occurred in three PAOs without any recanalization and recurrence. Likewise, Zhang et al. (21) report 93.3% nearly complete and complete occlusion rate of unruptured vertebral no-saccular aneurysm after PED placement in 32 cases series (21). Kuhn et al. (10) reported 100% nearly complete and complete occlusion rate in six patients with unruptured vertebral aneurysms. The similar results were reported by Yeung et al. (22). These data suggested that unruptured vertebral artery aneurysm may be effectively managed with PED. On the other hand, Stent assisted coiling is associated with high risk of incomplete occlusion and recurrence. Zhao et al. (12) reported 52.7% partial obliteration rate and 10.3% recurrence in 97 patients with vertebral artery aneurysm treated by stent assisted coiling, suggesting recurrence is closely associated with PICA involvement and immediate occlusion degree. Kim et al. (23) also reported 13% recurrence rate after conventional endovascular treatment of vertebrobasilar dissecting aneurysms, and concluded PICA involvement was the independent risk factor for recurrence. Despite the promising results in the present study, long-term angiographic follow-up and large study are needed to assess the efficacy of the PED in unruptured vertebral artery aneurysm involved PICA.

PAO is one of serious complications related to PED. Becsek et al. (24) reported 5 cases of PAO in their study for PED and concluded that non-compliance or resistance to antiplatelet therapy and severe in-stent stenosis might lead to PAO and evaluating antiplatelet effectiveness might be useful to reduce this complication. Oishi et al. (25) reported a patient who developed PAO during the 28 months follow-up after treatment with PED and found thrombus development at the non-covered part with endothelium due to discontinuation of antiplatelet and incomplete occlusion of the aneurysm. In the present study, PAO were developed in three cases during the follow-up. One patient developed PAO at 5 months due to the stenosis of the parent artery, another patient occurred PAO at 11 months attribute to discontinuation of antiplatelet at 6 months after procedure. We are not sure the mechanism of the last patient. There is no *in-situ* stenosis in the parent artery, the antiplatelet therapy was continued, and the deployment of PED was successful. A reasonable explanation might be the resistance of the antiplatelet medication. It might be useful to decline this complication with *in-situ* stenosis angioplasty, longer antiplatelet treatment and routinely monitoring antiplatelet effectiveness. Three PAOs resulted in obliteration of two involving PICA, fortunately, these patients did not develop any new neurological deficit, and there were no postoperative strokes in the treated PICA territory. This may due to slow progress and sufficient time for the establishment of collateral circulation.

The limitations of the current study include the retrospective property and variable follow-up intervals, which may cause selection bias of patients and add difficulties for replication. This study will provide some initial experience for the treatment of such lesions, while the result needs to be consolidated by large multi-center prospective studies.

## Conclusion

Our preliminary experience of using PED in the treatment of unruptured vertebral artery aneurysm involving PICA demonstrated that this method is effective and safe with favorable angiographic and clinical outcomes.

## Data Availability Statement

The raw data supporting the conclusions of this article will be made available by the authors, without undue reservation.

## Ethics Statement

The studies involving human participants were reviewed and approved by Medical Ethics Committee of Beijing Tiantan Hospital. Written informed consent to participate in this study was provided by the participants' legal guardian/next of kin.

## Author Contributions

WF, HJ, and YL designed, conceptualized the study, and analyzed and interpreted the data. WF, HJ, HG, GL, XM, and JW collected the data. WF drafted the manuscript. HJ, HG, GL, XM, JW, and YL revised the manuscript for intellectual content. All authors agreed and approved the publication.

## Conflict of Interest

The authors declare that the research was conducted in the absence of any commercial or financial relationships that could be construed as a potential conflict of interest.
